# Retreatment of a 6-Canalled Mandibular First Molar with Four Mesial Canals: A Case Report

**Published:** 2010-08-15

**Authors:** Mohsen Aminsobhani, Noushin Shokouhinejad, Sholeh Ghabraei, Behnam Bolhari, Abdollah Ghorbanzadeh

**Affiliations:** 1. Department of Endodontics, Dental School/Dental Research Center, Tehran University of Medical Sciences, and member of Iranian Center for Endodontic Research, Tehran, Iran.; 2. Department of Endodontics, Dental School/Dental Research Center, Tehran University of Medical Sciences, Tehran, Iran.

**Keywords:** First Mandibular Molar, Four Root Canal, Mesial Root

## Abstract

Successful root canal treatment requires adequate knowledge regarding morphologic variations in root canal system of teeth. This report describes a six-canalled mandibular first molar with four mesial root canals requiring endodontic retreatment. The two additional canals in the mesial root were found during retreatment with the aid of illumination and magnification. In conclusion, the possibility of atypical morphology and additional canals should never be overlooked.

## INTRODUCTION

One of the most important prerequisites for successful endodontic treatment is adequate knowledge and understanding of root canal anatomy. Numerous in vitro and in vivo studies have investigated the root canal morphology of mandibular first molars. Most of them had two roots, with two canals in the mesial root and one canal or two canals in the distal root [1][2][3]. The incidence of mesial roots with three root canals in mandibular molars has been reported to range between 1-15% [3][4][5][6][7][8][9][10]. Very few articles have demonstrated four canals in the mesial root of mandibular first molars [11][12][13].

This case report describes a six-canalled mandibular first molar with four canals in the mesial root, and two in the distal; this tooth had previously received incomplete root canal treatment.

## CASE REPORT

A 30 year old female with a non-contributory medical history was referred to our private office with severe spontaneous pain and tenderness to percussion in the right mandibular region. The patient reported that the tooth #30 had been root treated one month ago. She had experienced localized swelling and pain since then.

Clinical examination revealed extensive amalgam restoration in the mandibular molar; however no swelling or sinus tract was present. Tooth #30 was sensitive to palpation and percussion. There was no mobility and probing did not reveal the presence of a periodontal pocket. The adjacent teeth responded normally to thermal and electrical pulp testing, and to palpation, and percussion.

**Figure 1:**
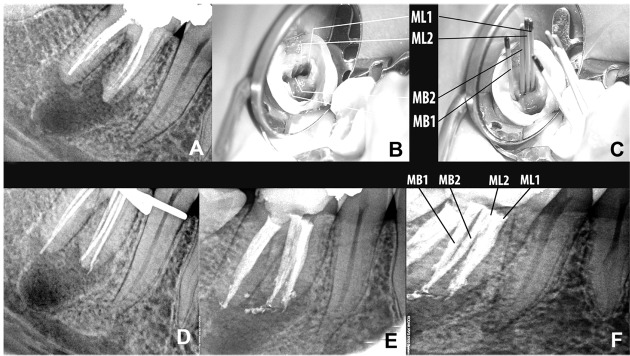
A) Preoperative radiograph B) Four root canal orifices can be observed: mesiobuccal 1 (MB1), mesiobuccal 2 (MB2), mesiolingual 1 (ML1), and mesiolingual 2 (ML2) C) Four gutta-percah master cones in four mesial canals D) Length confirmation of radiograph demonstrating four root canals in mesial root E) A radiograph after completion of retreatment. F) Postoperative radiograph showing four mesial canals in mesial root

Radiographic examination revealed a large radiolucency associated with the mesial root of tooth #30 (Figure 1A). A diagnosis of secondary or exacerbating apical periodontitis associated with incomplete root canal treatment was made. Therefore, endodontic re-treatment was indicated.

Local anesthesia with inferior alveolar nerve block using lidocaine (Daroupakhsh, Iran) was performed. After removing the coronal filling material and carious tooth structure, a prefabricated post was removed from the distal root. The tooth was isolated with a rubber dam and an adequately sized access cavity preparation was created. The floor of the pulp chamber was explored under magnification and illumination with the aid of a HEINE 2.5× HR Binocular Loupe and 3S LED HeadLight® (HEINE Optotechnik GmbH & Co. Herrsching, Germany). Two mesial and two distal root canal orifices had been inadequately treated during the first root canal treatment. Root filling materials were removed from the root canals with the aid of hand and rotary files. The unusually wide space between the two mesial orifices clinically and the radiographic appearance of the root canals indicated possible additional canal(s) in the mesial root. Moreover, during exploration of the groove with a sharp explorer and file, a sticky groove was felt. Two additional canals were located between the two previously discovered mesial orifices (Figure 1B). The working lengths of the root canals were determined using an electronic apex locator (ProPex™, Dentsply Maillefer, Montigny le Bretonneux, Switzerland) and were then confirmed with a digital radiography (Kodak RVG 5100 Digital Radiography System, Eastman Kodak Co., Trophy Radiology, Marne-la-Vallée, France). Root canal instrumentation was performed with ProTaper series Ni-Ti instruments (Dentsply Maillefer, Ballaigues, Switzerland) and EDTA gel (MD-ChelCream, Meta Biomed Co., Ltd., Cheongju City, Chungbuk, Korea). The root canals were irrigated with 2.5% sodium hypochlorite during instrumentation. Afterwards, the root canals were dressed with calcium hydroxide paste (Calcipex II, NISHIKA, Shimonoseki, Japan) and coronally sealed with a temporary filling material (Zonalin, Kemdent, UK).

At the next appointment (after 10 days), the tooth was asymptomatic. After completion of the chemomechanical preparation and radiographs with gutta-percha master cones (Figure 1C, Figure 1D), the root canals were dried with paper points and obturated with laterally compacted gutta-percha (Meta Biomed Co., Ltd., Cheongju City, Chungbuk, Korea) and AH26 sealer (Dentsply, DeTrey, Konstanz, Germany) (Figure 1E, Figure 1F). Coronal access was restored with a temporary filling material (Zonalin, Kemdent, UK) and the patient referred to another dentist for a coronal restoration and completion of the treatment.

**Figure 2:**
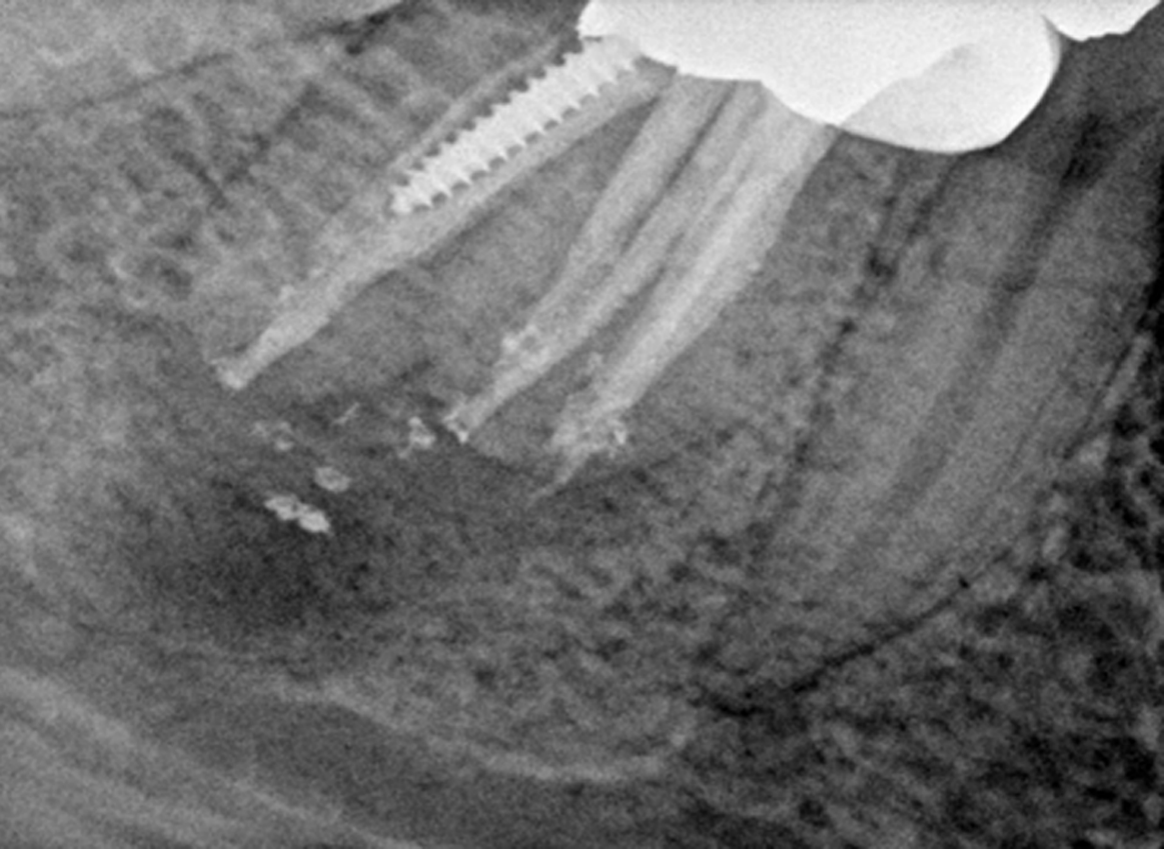
Three-month follow up radiograph

At the 3-months follow up examination, tooth #30 was functional and asymptomatic, with no clinical signs. There was radiographic evidence of periapical osseous healing (Figure 2). Further follow up examinations were recommended for evaluation of treatment outcome.

## DISCUSSION

Thorough understanding about root canal morphology plays an important role in the success of root canal therapy. Anatomical variations in root canal system should be considered when exploring the floor of the pulp chamber.

Multiple canals in the mesial root of mandibular molars have been reported in several cases [4][5][6][7][8][9]; however only two reports have described four canals in the mesial root of mandibular first molars [11][13]. Reeh described an interesting retreatment case of a seven-canalled mandibular first molar with four canals in the mesial root and three canals in the distal root [11]. Kontakiotis and Tzanetakis described four independent root canal orifices in the mesial root of a mandibular first molar with the aid of a dental operating microscope [13].

Exploration of the pulp floor groove under magnification and illumination revealed two further independent root canal openings between the already treated two mesial orifices. The two buccal canals merged into one foramen; the same was true for the two lingual canals (i.e. a total of 2 foramina in the mesial root).

Meticulous radiographic evaluation of the root and periodontal ligament outlines can help to predict unusual anatomy. Moreover, the use of a sharp explorer, as well as the additional methods such as troughing of the grooves with ultrasonic tips, magnification and illumination can assist to locate extra root canal orifices [14][15][16].

## CONCLUSION

In conclusion, although detecting extra canals in teeth with atypical internal anatomy is challenging, repeated attempts should be made to find all root canals.
